# Prevalence of engagement and frequency of non-suicidal self-injury behaviors in adolescence: an investigation of the longitudinal course and the role of temperamental effortful control

**DOI:** 10.1007/s00787-022-02083-7

**Published:** 2022-09-19

**Authors:** Concetta Esposito, Mirella Dragone, Gaetana Affuso, Anna Lisa Amodeo, Dario Bacchini

**Affiliations:** 1 Department of Humanities, University of Naples “Federico II”, Naples, Italy; 2 Department of Psychology, University of Campania “L. Vanvitelli”, Caserta, Italy

**Keywords:** Non-suicidal self-injury, Effortful control, Developmental trajectories, Adolescence

## Abstract

Non-suicidal self-injury (NSSI) is among the most frequent maladaptive behaviors reported in adolescence, with prevalence rates around 18–22% in community samples worldwide. The onset of NSSI typically occurs between early and middle adolescence, with a peak during middle adolescence (14–15 years) and a subsequent decline during late adolescence. This study investigated the growth curves of NSSI across four years during adolescence, analyzing simultaneously the probability of engagement in NSSI at least once (i.e., prevalence) and the frequency of engagement once initiated (i.e., severity). Furthermore, the study examined the predicting role of effortful control on NSSI over time (time-varying effects), net of other key risk factors for NSSI, such as anxiety-depression and bullying victimization. A sample of 430 Italian adolescents enrolled in Grade 9 at baseline was involved in the study. Based on the Latent Growth Curve Zero-inflated Poisson methodology, the results indicated a negative quadratic trend of both NSSI prevalence, with an increase between T1 and T3 followed by a decrease in the subsequent wave, and NSSI frequency once initiated, with a peak at T2 followed by a decline over time. The results also showed that adolescents who reported low effortful control abilities had a heightened probability of involvement in NSSI at each time point, whereas no significant association was found with NSSI frequency once initiated. Findings from this study offer important insights into the developmental course of NSSI and point out the need for future in-depth investigations of the mechanisms that might underlie NSSI prevalence and severity throughout adolescence.

**Supplementary Information:**

The online version contains supplementary material available at 10.1007/s00787-022-02083-7.

## Introduction

Non-Suicidal Self-Injury (NSSI) is defined as the direct and deliberate infliction of damage on one’s body tissue without suicidal intent and for purposes that are not culturally accepted [1]. While NSSI was only listed as a symptom of Borderline Personality Disorder in DSM-IV [2], research during the past decades has led to the inclusion of NSSI as an independent diagnostic entity in DSM-5 [3]. This has encouraged NSSI researchers to correct several misconceptions regarding its initial diagnosis and further investigate the individual factors and relational patterns associated with this behavior, also in non-clinical samples [4, 5]. 

Meta-analytic research has pointed out that approximately 18–22% of adolescents from community samples have engaged in this kind of behavior at least once in their life [6, 7]. As regards the 12-month prevalence rate for NSSI during adolescence, based on a collection of studies from 2011 to 2018 (mean age of participants between 15 and 16 years), Lim et al. [7] concluded that 19.5% of the total sample have engaged in NSSI. Similarly, Gillies et al. [8] analyzed prevalence rates of NSSI in community-based studies from 1990 to 2015, finding that the past-year rate for adolescents aged 12 to 18 years was 18.6%. In general, rates of adolescents who meet diagnostic criteria of NSSI frequency according to DSM-5 (≥ 5 instances in the past year) are generally lower and fall between 1.5% and 6.7% [9].

Secondary research including a systematic review of the literature [10], based on short-term longitudinal investigations (average length of follow up = 20 months), and a pooled event history analysis [11], has suggested that the prevalence rates of NSSI typically peak during early- to mid-adolescence (14–15 years), followed by a decline through late adolescence and early adulthood (around 18 years). However, the developmental course of NSSI remains not fully understood as only a few studies, to date, have addressed this point from an extended longitudinal perspective. Using an accelerated 10-year longitudinal study of 662 Canadian adolescents aged 12 to 18 years at baseline, Turner et al. [12] confirmed what emerged from previous secondary research [10, 11]. More in detail, the authors found that adolescence (from 12 to 17 years) was a critical stage for NSSI initiation, whereas early adulthood (ages 18–21) represented a typical period for NSSI cessation. Similarly, in the study by Daukantaité et al. [13], 1064 students from Southern Sweden, enrolled in grades 7 and 8 at baseline (participants’ mean age = 13.7 years), were followed one year and 10 years later. The findings showed that the prevalence of NSSI (at least one instance) significantly declined from 40% in adolescence to 18.7% in early adulthood.

Although these studies have significantly advanced our knowledge of the NSSI development over time, they included a number of limitations that may have influenced the results. First, many studies primarily relied on samples of participants with a wide range of ages at each time-point of the data collection [e.g., 14, 15]. Further, the use of different assessment methods across studies (e.g., yes/no questions or counts of occurrences of NSSI) often makes it difficult to compare the findings from different research, thus preventing from building a comprehensive understanding of the longitudinal course of NSSI. Furthermore, only in a few studies [e.g., 16], appropriate statistical approaches to handle the bound excessive number of zeros in the NSSI data (around 80–90%; [17]) have been applied. You et al. [15], for instance, assessed NSSI trajectories in a sample of adolescents aged 12 to 18 years at Time 1, finding a downtrend curvature of the NSSI frequency over three consecutive semesters (spring, fall, and spring semesters). NSSI was rated on a 4-point scale (from 0 = ‘never’ to 3 = ‘six times or more), and Latent Growth Curve Analysis [18] was performed, apparently without accounting for overdispersion or zero-inflation in the data. Conversely, participants in the study by Wan et al. [14] were from grade seven of junior school to grade two of the college. NSSI was treated as a dichotomous variable (frequency of NSSI ≥ 1, ‘yes’ as opposed to ‘no’) and only descriptive statistics of NSSI engagement for each year were reported, indicating, also in this case, a decline over time (from 17 to 8.8% after six months).

According to the theoretical model of NSSI proposed by Nock [1], people use NSSI as a maladaptive strategy to cope with stressful events, which allows them to both regulate their emotional/cognitive experiences and communicate with or influence others. This strategy arises from intra- (e.g., elevated physiological arousal) or inter-personal (e.g., deficits in social problem solving and communication) vulnerabilities that predispose them to perceive such events as particularly overwhelming and difficult to handle [19, 20]. Despite the considerable heterogeneity between the studies, such as differences in the definitions of NSSI, sample recruitment and selection, and study design, a wide variety of stressful life events such as bullying victimization [21–23] and several psychopathological conditions, such as depression or anxiety [24–26] have been consistently associated with NSSI, also longitudinally [12, 27–29]. In an attempt to identify intrapersonal vulnerability factors that might contribute to the use of NSSI, other studies have related NSSI with personal characteristics that define individual differences, examining, for instance, the role of personality traits [e.g., 30, 31], or self-regulation abilities such as impulsivity [32–34] and temperamental effortful control [35, 36]. Indeed, it is generally acknowledged that temperament plays a crucial role in the onset and maintenance of behavior problems in adolescence [37, 38]. Based on the temperament model of Rothbart [39], effortful control (EC) encompasses individuals’ abilities to effortfully modulate their thoughts, emotions, and behavior. It is genetically influenced, biologically based, and shaped by socialization and contextual experiences [40]. One of the functions of EC is to regulate negative reactivity through the regulation of attention and the inhibition of automatic cognitive and behavioral responses [41], thus reducing the likelihood of emitting inappropriate or undesirable responses as a result of high negative reactivity. Based on the theory and previous evidence suggesting that NSSI works as an emotion regulation strategy aiming at reducing negative emotionality [42, 43], it is possible that low effortful control might serve as an intrapersonal vulnerability factor for NSSI engagement. Built on this model, Baetens et al. [35] investigated EC differences between community adolescents with and without NSSI, finding that those who endorsed NSSI also displayed lower levels of EC than adolescents without NSSI. The study by Raemen et al. [36] supported the hypothesis that EC is significantly associated with NSSI, but their sample included community adults aged between 19 and 64 years. Noteworthy, Cassels et al. [44] outlined the importance of examining the predictive role of self-regulation abilities on NSSI over and above the effects that other risk factors might have, such as psychological distress, depression, anxiety, and childhood trauma. As the authors stated, empirical research is needed to clarify if self-regulation is itself a significant predictor of NSSI, or if it is simply associated with other more meaningful antecedents of NSSI. Overall, the findings from their study provided evidence that deficiencies in self-regulation were a significant predictor of new-onset of NSSI, independently of general distress. Longitudinal studies with multiple risk factors are needed to fully establish the nature of this relationship.

In light of the above considerations, this study had two main goals. First, it aimed to examine the longitudinal trajectories of NSSI across four years, specifically taking care of disentangling change related to NSSI engagement at least once (i.e., prevalence) and change of the frequency of NSSI episodes over time once initiated (i.e., severity). More specifically, zero-inflated models were used to handle the NSSI data. These models combine two distributional forms, one for the binary component (‘no’ vs. ‘yes’ responses) and one other for the count model component (i.e., the frequency of occurrences). The theoretical assumption of zero-inflated models is that zero responses may come from a person who has never engaged in NSSI (also referred to as structural zeros) or from a person who engages or has engaged in NSSI in the past but did not within the specific time frame covered by the research tools for NSSI screening (also referred to as random zeros; [45]). Using zero-inflated models, this study considered the possibility that there are multiple types of adolescents, those who engage in NSSI more or less regularly and those who do not currently engage.

As a second aim, this study investigated the predictive role of EC on NSSI over time. As suggested by previous literature, the contribution of EC was examined as independent of the effects of other key factors that have been consistently linked to NSSI longitudinally [29], which are anxiety-depressive symptoms and experiences of bullying victimization. Furthermore, we explored the role of adolescent gender in predicting NSSI change over time. To date, previous studies have examined the effect of gender on NSSI prevalence, finding mixed results. Some studies evidenced that females generally engage in NSSI more than males [e.g., 26, 29], whereas others reported equivalent NSSI prevalence rates [6]. In terms of NSSI trajectories throughout adolescence, the study by Barrocas et al. [16] indicated that, although decreasing over time, males were more likely to report a higher frequency of NSSI episodes compared to females.

Finally, to enhance the longitudinal validity of the study, age imbalances in the sample were greatly limited by involving only a cohort of regular students who were enrolled in the same grade at baseline. More in detail, based on the literature above discussed [10, 11] that stressed the relevance of mid-and late adolescence for NSSI, this study focused on a sample of adolescents who were recruited between early and mid-adolescence (13–14 years) and followed until the beginning of late adolescence (16–17 years). Overall, we hypothesized that NSSI prevalence over time followed a downward trend, with a peak at approximately 15 years of age. Furthermore, since there is little and mixed evidence in the literature about how the frequency of NSSI behavior changes over time, no hypotheses were advanced in this case. Similarly, while we expected that EC, anxiety-depression, and bullying victimization were significantly and independently associated with NSSI prevalence at each time point, we had no specific hypotheses regarding their effects on NSSI frequency over time.

## Method

### Participants and procedure

The sample consisted of 430 Italian adolescents attending, at the first data assessment, the 9th grade of vocational and academic high schools (47.9% males, *M*_age_ at Time 1 (T1) = 14.18, SD = 0.55). The sampling procedure did not utilize a full probability sampling design. Participants were recruited from university-school collaborative networks in Naples, in Southern Italy. Most of them were from two main high schools (38.9 and 31.6%), whereas the remaining proportion came from 23 other schools in the same geographic area. The schools were situated in low- or medium-income areas. As for the sociodemographic composition of the sample, more than half of the participants’ parents only completed elementary or middle school (59%); 49% of fathers were unemployed or did a lower-skilled job, whereas 66.3% of mothers were homemakers. In general, the socioeconomic distribution of participants’ families matched the Italian national statistics [46].

The study was conducted in compliance with the Code of Ethics of the Italian Association of Psychology, the Code of Ethics of the National Order of Psychologists, and Helsinki’s Declaration and was approved by the university review board and authorities of schools involved in the research. After the first data collection (spring 2016), participants were recontacted every year for four consecutive times (until spring 2019). Data collections were conducted by trained research assistants in schools during regular class hours. Students who were absent during the first collective administration of the questionnaires were invited to complete the questionnaire at a later date. Recruitment letters describing the study were sent home to obtain the parents’ written consent for the child to participate in the study.

### Measures

#### Non-suicidal self-injury

NSSI for each time point was assessed through a six-item scale [26, 47] measuring how frequently (0 = ‘never’; 1 = ‘1–2 times’; 2 = ‘3–5 times’; 3 = ‘6–9 times’; 4 = ‘10 or more times’), during the last six months, adolescents intentionally engaged in several types of self-injurious behaviors without suicidal intentions (such as cutting, burning, or hitting oneself). A composite score of NSSI for each time was obtained by summing the frequency rating for each item, in line with prior research [26, 47], with higher values indicating a higher frequency of engagement in NSSI. This measure has been previously administered to adolescent samples, including Italian adolescents [22, 26], and has shown good reliability and convergent validity [47]. In the current sample, Cronbach’s alphas and McDonald’s Omega coefficients ranged from 0.83 to 0.90, thus indicating good internal consistency of the measure.

#### Effortful control

Temperamental EC was assessed using 21 items from the long version of the Early Adolescent Temperament Questionnaire-Revision (EATQ-R) [48]. Each item was rated on a 5-point scale (from 1 = ‘almost never true’ to 5 = ‘almost always true’), and a composite score was obtained by averaging all items. Sample items were “I pay close attention when someone tells me how to do something” and “When someone tells me to stop doing something, it is easy for me to stop.” Previous studies have supported the psychometric soundness and validity of this measure in Italian samples [49, 50]. In the current study, Cronbach’s alphas and McDonald’s Omega coefficients ranged from 0.80 to 0.86 and from 0.81 to 0.86 across all assessments, thus indicating a good level of reliability.

#### Bullying victimization

The measure of victimization was collected by using an adapted version of the Bully-Victim Questionnaire by Olweus [51]. Participants responded to 9 items rated on a 5-point scale concerning how frequently (from 1 = ‘never’ to 5 = ‘several times a week’), during the last six months, they had been victimized through verbal, physical or relational behavior. A composite score was computed by averaging all items, with high scores indicating higher victimization. Sample items were “Other students called me names,” “I have been physically attacked by other students,” and “Other students excluded me from social activities (e.g., parties, sports activities, etc.)”. A detailed definition of bullying victimization was included in the questionnaire, emphasizing the intention to harm the victim, the repetitive nature of episodes, and the imbalance of power between the victim and the perpetrator(s) as the three key elements of bullying. Confirmatory factor analyses with item parceling showed perfect model fit (CFIs = 1.00, RMSEAs = 0.00, SRMRs = 0.00), thus supporting the psychometric properties of the instrument. Cronbach’s alphas and McDonald’s Omega coefficients ranged from 0.81 to 0.88 and from 0.79 to 0.86, thus providing further evidence of the adequacy of the scale in the Italian context [26, 52].

#### Anxiety-depression

Symptoms of anxiety-depression were measured using the Youth Self Report Questionnaire (YSR 11/18) [53]. Adolescents were asked to report whether they had experienced symptoms of anxiety or depression (e.g., feeling lonely, crying a lot) in the past six months using a three-point scale (0 = ‘not true’, 1 = ‘somewhat or sometimes true, or 2 = ‘very true or often true’). A global score of anxiety-depression was obtained by averaging the score of all items on the scale for each participant. Previous studies have supported the validity of this measure in many samples from many cultures (e.g., [54]), including Italy [55]. Internal consistency for this sample was good for all time-point assessments, with Cronbach’s alphas and McDonald’s Omega coefficients ranging from 0.87 to 0.88.

#### Analytic plan and preliminary analyses

Latent growth curve (LGC) [18] analyses were conducted in Mplus 8 [56]. Missing data were handled using the full information maximum likelihood (FIML) method with the assumption that the data were missing at random. To choose the most appropriate solution for modeling change with the NSSI outcome, the proportion of zero in the NSSI measures, the means and variances.

were examined to detect the nature and degree of overdispersion in the data.

To model over dispersed data, negative binomial (NB), zero-inflated Poisson (ZIP), and zero-inflated negative binomial (ZINB) distributions would be appropriate, depending on the degree of overdispersion, the presence of zero-inflation, and the relationship between zero-inflation and overdispersion. The NB model is appropriate for the analysis of highly skewed and over-dispersed count data, without the zero-inflation. The ZIP model uses the Poisson distribution to account for the excess of zeros [57], and to address some of the variation resulting from overdispersion in the outcome variable due to zero-inflation. The ZINB model is utilized when data are over-dispersed, above and beyond the context of the excess zeros present in the data. ZIP and ZINB are similar to one another, except that ZINB includes an extra parameter that accounts for overdispersion unrelated to excess zeros. The advantage of the ZINB model is that it can account for both the overabundance of zeros and a greater level of overdispersion in the outcome than the ZIP model. To choose the most adequate model to analyze the count data of zero observation, we compared the only-intercept (postulating no change over time) ZIP and NB models with ZINB. First, the significance of the overdispersion parameter in NB and ZINB was evaluated to detect the presence of overdispersion in the data and determine whether overdispersion was related to excess zeros or the general outcome variable. Information criteria (Akaike Information Criterion [AIC], the Bayesian Information Criterion [BIC], and the sample size adjusted BIC [A-BIC]) were used to evaluate the alternative models in terms of statistical fit. In general, lower values indicate a better fitting model.

**Fig. 1:**
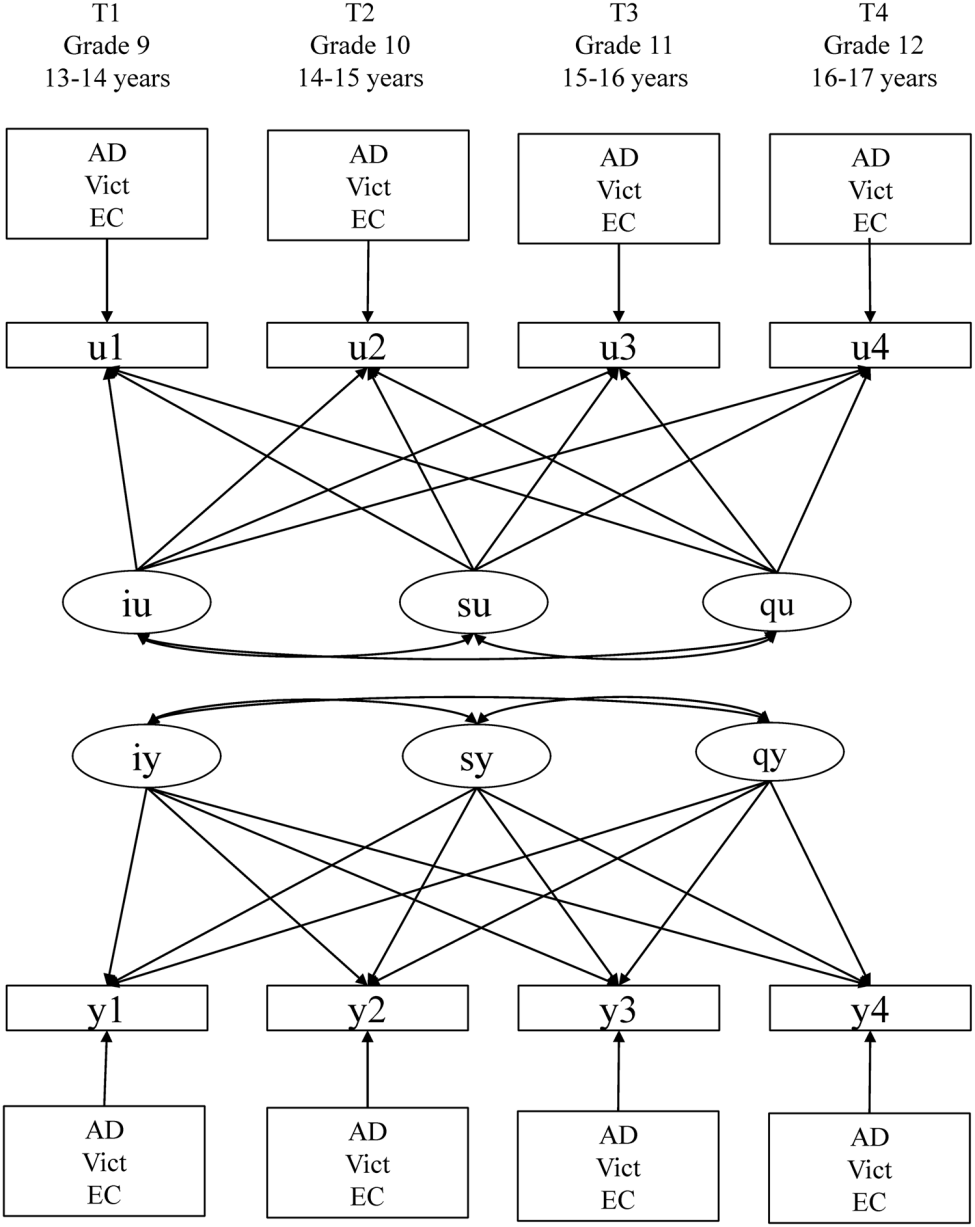
The hypothesized Latent Growth Curve Zero-inflated Poisson Model. *U-part* zero-inflated part (engagement vs. no engagement); *Y-part* count part (frequency of engagement once initiated). *AD* anxiety-depression symptoms; *Vict* bullying victimization; *EC* effortful control

Once identified the most appropriate model, two additional two-factor (intercept, slope) LGC models were run and compared with the only-intercept model (also referred to as unconditional models). In the first model, a significant linear change over time was postulated (i.e., intercept and linear slope); in the second model, a non-linear significant change over time was assumed (intercept, linear and quadratic slope). The fit of these three competing models was evaluated by examining the parameter estimates and compared using the likelihood ratio test and the Information Criteria [58]. After establishing the best fitting growth curve model, adolescent gender (1 = male, 2 = female) was included as a predictor of the growth curve factors (intercept and slopes), whereas effortful control, anxiety-depression, and bullying victimization measures were included as time-varying covariates that directly influenced repeated measures of NSSI (see Fig. 1).

**Fig. 2:**
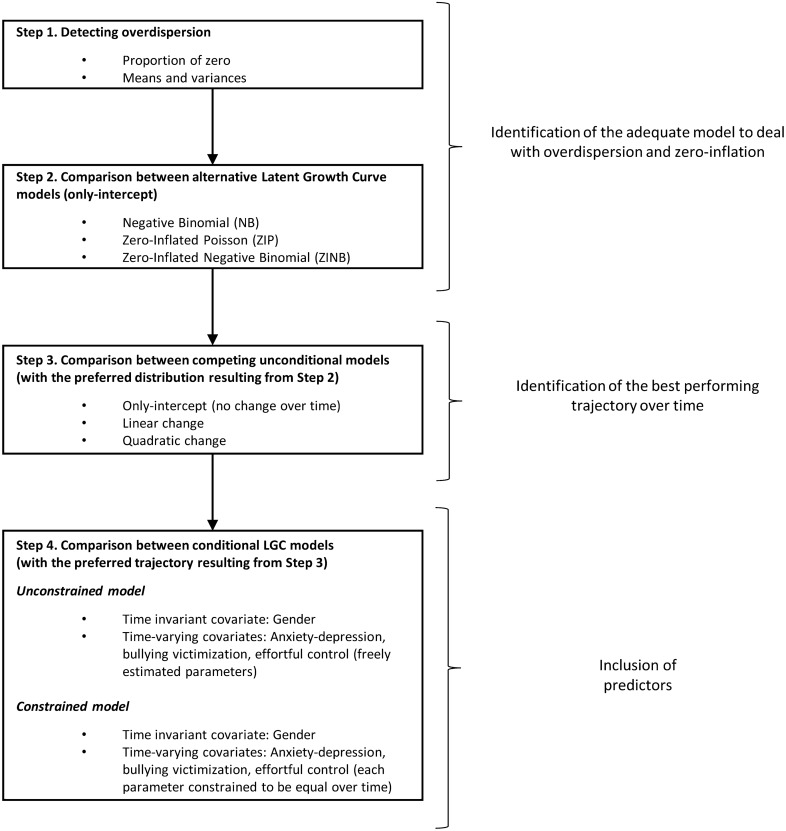
The flowchart of the analytic plan

Finally, to establish whether the effects of effortful control, anxiety-depression, and bullying victimization on NSSI repeated measures were invariant over time, an additional model in which the effect of each variable was constrained to be equal across time was performed and compared with the baseline model in which the parameters were freely estimated. The fit of these alternative models was compared using the likelihood ratio test. The flowchart of the analytic plan is illustrated in Fig. 2.

## Results

### Missing data and attrition analysis

The overall attrition rate of the study was 13%, with 374 participants providing data at all four assessment points. More in detail, all T1 participants completed the measures at Time 2 (T2). Of these, 32 were missing at Time 3 (T3; 7.4%) and 24 more (5.6%) at Time 4 (T4). The loss of participants over time was mainly due to participants dropping out of school. The Little’s test [59] for data missing completely at random (MCAR) in SPSS 21 was significant, *χ*^2^ (20) = 37.67, *p* < 0.05, suggesting that missingness was not completely at random. Independent t-tests comparing mean differences between missing and non-missing cases indicated that those who dropped out from the study reported lower levels of EC at all previous time points (all *p*s < 0.05). Furthermore, missingness on all the study’s variables at T4 was dependent on levels of NSSI at T3, with those who were missing at T4 reporting higher levels of NSSI at T3, *t* (105,3) = 3.1, *p* < 0.01. In terms of descriptive statistics, 10 participants among those who reported having engaged in NSSI at least once at T1 dropped out of the study at T3, and two more at T4 (total percentage of missing cases = 2.8%). However, of those who dropped out at T3, only 4 reported having engaged in NSSI also at T2, whereas no participants of those who dropped out at T4 reported NSSI also at T2 and T3. Overall, since the outcome scores for dropouts tended to be correlated with previously recorded responses from earlier assessment points, a missing at random (MAR) [59] pattern was assumed.

### Descriptive statistics

**Fig. 3:**
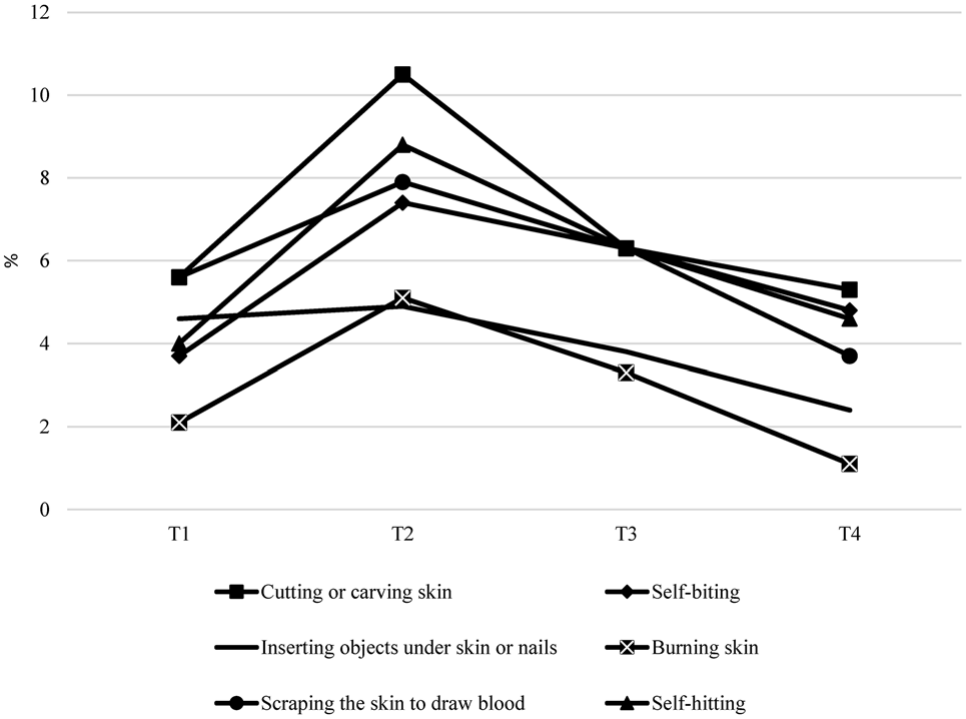
Percentages of adolescents who engage in each specific NSSI behavior across time points

On average, the percentage of adolescents who had engaged in NSSI behavior across the four time-point was approximately 12%. Chi-squared tests revealed no significant differences in prevalence rates of NSSI engagement between females and males (all *p*s > 0.05). Adolescents who engaged in NSSI at T1 were 45 (10.5% of the total sample). Of these, 21 reported having engaged in NSSI at least once also at T2 (approximately 47% of T1 NSSI participants), and 12 at both T2 and T3 (note that at T3, 10 of T1 NSSI participants dropped out of the study). This number declined at T4, with only 8 adolescents continuing reporting NSSI behavior. The percentages of adolescents who engaged in severe NSSI (six times or more), based on the total sample, were 6.5% at T1, 7.7% at T2, 5% at T3, and 3.7% at T4. The percentage of new-onset of NSSI was 9.1% at T2, 6.6% at T3, and 3% at T4. For each specific NSSI behavior assessed in the study, the average prevalence rate across the four waves was 7% for cutting or carving skin (range = 5.3% at T4–10.6% at T2), 5.6% for self-biting (range = 3.7% at T1–7.4% at T2), 3.9% for inserting objects under skin or nails (range = 2.4% at T4–4.9% at T4), 2.9% for burning skin (range = 1.1% at T4–5.1% at T2), 5.9% for scraping the skin to draw blood (range = 3.7% at T4–7.9% at T2) and self-hitting (range = 4% at T1–8.8% at T2; see Fig. 3).

The bivariate correlations among NSSI and the other study’s variables are reported in Table 1. As expected, NSSI was positively associated with anxiety-depression, both concurrently and longitudinally. Significant associations were found between cross-sectional measures of NSSI and EC, with low levels of EC associated with higher NSSI values, and between NSSI and bullying victimization, with higher victimization associated with higher NSSI at each time point but T4.

**Table 1:** Correlations among the study’s variables

	1	2	3	4	5	6	7	8	9	10	11	12	13	14	15	16
1. NSSI T1	1															
2. NSSI T2	0.22***	1														
3. NSSI T3	0.13**	0.29***	1													
4. NSSI T4	0.31***	0.24***	.41***	1												
5. EC T1	– 0.09*	– 0.11**	– 0.09	– 0.08	1											
6. EC T2	0.00	– 0.13***	– 0.10*	– 0.05	0.67***	1										
7. EC T3	– 0.12**	– 0.11**	– 0.13**	– 0.14**	0.60***	0.65***	1									
8. EC T4	– 0.08	– 0.11**	– 0.14**	– 0.11*	0.66***	0.73***	0.76***	1								
9. Anxiety-depression T1	0.28***	0.21***	0.13**	0.15***	– 0.26***	– 0.16***	– 0.45***	– 0.14***	1							
10. Anxiety-depression T2	0.15**	0.37***	0.16***	0.21***	– 0.26***	– 0.23***	– 0.23***	– 0.23***	0.61***	1						
11. Anxiety-depression T3	0.17***	0.27***	0.30***	0.26***	– 0.28***	– 0.23***	– 0.31****	– 0.27***	0.46***	0.57***	1					
12. Anxiety-depression T4	0.18***	0.16**	0.15^**^	0.33***	– 0.27***	– 0.20***	– 0.34***	– 0.27***	0.42***	0.48***	0.65***	1				
13. Bullying Victimization T1	0.19***	0.18***	0.20***	0.15**	– 0.17***	– 0.15***	– 0.19***	– 0.18***	0.31***	0.23***	0.32***	0.22***	1			
14. Bullying Victimization T2	0.06	0.19***	0.17***	0.08	– 0.20***	– 0.23***	– 0.19***	– 0.21***	0.25***	0.30***	0.35***	0.29***	0.36***	1		
15. Bullying Victimization T3	0.07	0.12*	0.27***	0.21***	– 0.18***	– 0.17***	– 0.17***	– 0.17***	0.12*	0.17***	0.29***	0.25***	0.20***	0.28***	1	
16. Bullying Victimization T4	0.07	0.12*	0.08	0.06	– 0.20***	– 0.17***	– 0.19***	– 0.16***	0.11*	0.16**	0.25***	0.34***	0.24***	0.33***	0.36***	1

^***^p < 0.001, ^**^p < 0.01, ^*^p < 0.05

### Latent growth curve models

#### Preliminary analyses

As displayed in Table 2, the proportion of zero in the NSSI measures across time ranged from 0.86 to 0.91. Overall, the variances (*σ*^2^ range = from 1.93 to 8.29) were greater than the means at each time-point (*M* range = from 0.33 to 0.87), thus indicating that NSSI measures were over-dispersed.

**Table 2:** Statistical Descriptive Information of NSSI Variables in the Analysis

	*M*	SD	Variance	Skewness (SE)	Proportion of zero
Time 1	0.52	2.099	4.404	6.095 (0.118)	0.895
Time 2	0.87	2.879	8.289	3.971 (0.118)	0.860
Time 3	0.53	1.916	3.669	5.117 (0.122)	0.867
Time 4	0.33	1.388	1.925	5.726 (0.126)	0.912

NSSI was measured using six items rated on an ordinal scale ranging from 0 (never) to 4 (10 or more times)

To choose the most adequate model to analyze the count data of zero observation, we assessed the model fits of only-intercept (postulating no change over time) ZIP, NB, and ZINB models.

As can be observed in Table 3, AIC, BIC and A-BIC were lower for ZINB compared to ZIP and NB. However, the dispersion parameter calculated by NB and ZINB was significant in NB but not in ZINB, thus suggesting that (i) data were over-dispersed (as indicated by the significant dispersion parameter in NB) and that (ii) this over-dispersion might be accounted only for the overabundance of zeros (as indicated by the non-significant dispersion parameter in the ZINB model). Thus, ZINB was excluded from the comparison. Although the NB model can also effectively deal with some degrees of zero-inflation, it cannot provide information about possible mechanisms underlying zero-inflation, as it only considers the count component of the model, thus excluding the binary component (‘yes’ vs. ‘no’ responses). Conversely, ZIP models examine two processes simultaneously: The probability of occurrence of NSSI (i.e., whether or not an individual engages in NSSI) and how often this behavior is expressed once initiated. Then, the ZIP model was finally preferred to NB and ZINB for further analyses.

**Table 3:** Model fits of NB, ZIP, and ZINB models – Only-intercept model

	ZIP	NB	ZINB
Log-Likelihood	– 1020.171	– 1010.526	– 995.527
AIC	2048.342	2027.052	2001.054
BIC	2064.597	2039.243	2021.373
A-BIC	2051.903	2029.723	2005.506
Dispersion parameter (*p*-value)		3.846, *p* < 0.001	0.585, *p* = 0.112

*AIC* Akaike Information Criterion, *BIC* Bayesian Information Criterion, *A-BIC* sample size adjusted BIC

#### Unconditional latent growth curve models

Table 4 presents the results of the unconditional ZIP growth curve models (i.e., linear and quadratic models). From the fit statistics and the examination of the model parameters, it was evident that the quadratic model was the most appropriate (lower AIC, BIC, and A-BIC) and then was preferred over the linear model.

**Table 4:** Model fit information and growth parameters for the unconditional ZIP models

	Only-intercept	Linear	Quadratic
Fit Indices			
AIC	2048.342	2044.354	2006.755
BIC	2064.597	2084.992	2079.903
A-BIC	2051.903	2053.257	2022.781
Log-Likelihood		– 1012.177	– 985.377
Parameters			
Binary outcome			
Means			
Intercept		3.150, *p* < 0.001	8.915, *p* < 0.001
Slope: Time (linear effect)		0.062, *p* < 0.001	– 10.321, *p* < 0.001
Slope: Time (quadratic effect)			3.870, *p* < 0.001
Variance			
Intercept		5.788, *p* < 0.05	66.809, *p* < 0.001
Slope: Time (linear effect)		0.003, *p* = 0.923	119.211, *p* < 0.001
Slope: Time (quadratic effect)			16.981, *p* < 0.001
Continuous outcome			
Means			
Intercept		1.089, *p* < 0.001	0.871, *p* < 0.001
Slope: Time (linear effect)		– 0.099, *p* = 0.357	0.606, *p* = 0.140
Slope: Time (quadratic effect)			– 0.305, *p* < 0.05
Variance			
Intercept		1.273, *p* < 0.01	1.251, *p* < 0.05
Slope: Time (linear effect)		0.120, *p* < 0.05	2.264, *p* < 0.01
Slope: Time (quadratic effect)			0.334, *p* < 0.01

*AIC* Akaike Information Criterion, *BIC* Bayesian Information Criterion, *A-BIC* sample size adjusted BIC

**Fig. 4:**
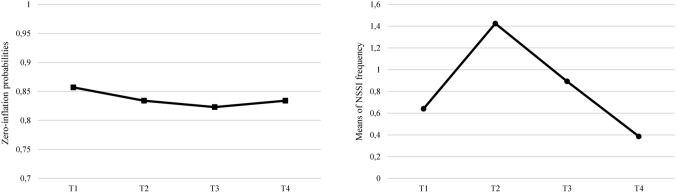
Trajectories of zero-inflated probabilities and mean frequency of NSSI over time

The parameter estimates for both the zero-inflated and count parts of the model are displayed in Table 4. As for the zero-inflated part of the model, the intercept and both the linear (negative effect) and quadratic (positive effect) slopes were significant, meaning that (i) there were significant inter-individual differences in the probability to engage in NSSI at baseline and that (ii) the probability of engagement in NSSI increased in the first part of the curve and then decreased in the last part. Consistently with the model growth parameters, the estimated probability for an individual to be in the zero class (no engagement in the last six months) at baseline was 0.857. Otherwise stated, 85.7% of the sample had structural zero at baseline. This probability decreased at T2 (0.834) and T3 (0.823) and slightly increased again at T4 (0.834). Taken together, these results suggested that there was significant inflation in the number of individuals who engaged in NSSI behavior during T2 and T3 and that this number significantly decreased at T4 (see Fig. 4).

The results for the count growth curve indicated a negative quadratic trend in the change of NSSI frequency of occurrences over time. That is, once initiated, the frequency of engaging in NSSI behavior firstly increased and then dramatically decreased (see Fig. 4). More specifically, exponentiating the intercept values yields 2.39, which was the expected value associated with NSSI behavior at baseline (from 3–5 times to 6–9 times, approximately) for individuals who engage in NSSI (individuals who were not structural zeros). The linear slope was not significantly different from zero. The quadratic slope was significant; exponentiating this value yields 0.73, which is the expected value of NSSI behavior (around 1–2 times) from approximate age 13 through age 17 for people who engage in NSSI. Lastly, we found significant covariances between the growth parameters in the zero-inflated part. More in detail, the zero-inflated intercept was positively associated with the quadratic slope (*p* < 0.001), implying that decreases in NSSI tend to occur at slower rates for those individuals who were more likely to engage in NSSI at baseline.

#### Conditional latent growth curve model

All the hypothesized effects of covariates were estimated for both the binary and count components of the LGC model (see Table S1 in supplemental materials for further details). The results indicated that the constrained model with the effects of each time-varying variable constrained to be equal over time performed better than the freely estimated model, Δ*χ*^2^ (23) = 39.15, *p* < 0.05. Specifically, EC was positively associated with the NSSI zero-inflated part, suggesting that high levels of EC significantly predicted the probability of being in the zero class at each time point (i.e., no engagement in NSSI; *B* = 0.79, *p* < 0.05). On the other side, anxiety-depression and bullying victimization had a significant negative effect on the binary NSSI outcome (Bs =  – 3.09 and – 0.66, *p*s < 0.001 and 0.5, respectively), meaning that higher levels of both significantly decreased the probability to have structural zeros. Stated in other words, higher levels of anxiety-depression and bullying victimization were associated with a heightened probability of engaging in NSSI at least once, and this effect was invariant over time. As for the count part of the model, only anxiety-depression had a significant and independent effect on the frequency of NSSI behavior once initiated (*B* = 0.64, *p* < 0.01). Neither EC (*B* =  – 0.18, *p* = 0.48) nor bullying victimization (*B* = 0.00, *p* = 0.98) were significantly associated with NSSI frequency of engagement. Finally, adolescent gender had no significant effects on both the zero-inflation and count intercepts, indicating no differences between males and females at baseline, both in terms of prevalence and frequency of engagement in NSSI. A significant effect was found on the zero-inflated linear (*B* =  – 2.005, *p* < 0.001) and quadratic slopes (*B* = 2.005, *p* < 0.001), suggesting that males had a higher probability of being in the zero-class over time, compared to females. No significant differences were found in the growth parameters of the count part of NSSI (all *p*s > 0.05), meaning that the frequency of engagement in NSSI once initiated was equal across gender.

## Discussion

This study examined the course of NSSI in a community sample across four years during adolescence. Overall, prior research has indicated a downward trend in NSSI rates [13] after a peak during early- to mid-adolescence (14–15 years) [10–12]. However, our understanding of the developmental trajectories of NSSI remains limited due to several methodological issues that might have influenced previous results or made it hard to draw comparisons between different studies. The current research sought to extend prior work by investigating the longitudinal course of NSSI disentangling the change related to NSSI engagement at least once (i.e., prevalence) and the change of the frequency of NSSI episodes over time once initiated (i.e., severity; Aim 1). Furthermore, drawing on previous literature analyzing individual differences as markers of risk for NSSI [35, 36], this study investigated the role of temperamental effortful control in predicting NSSI over time, net of other risk factors such as anxiety-depression and bullying victimization (Aim 2). In an exploratory perspective, the role of gender on NSSI trajectories over time was examined.

Based on descriptive statistics, the average percentage of adolescents who had engaged in NSSI across the four time-point was 12%, with prevalence rates being higher (14%, approximately) at T2 (Grade 10; 14–15 years) and T3 (Grade 11; 15–16 years). This percentage is relatively consistent with previous research involving non-clinical samples of adolescents [e.g., 6, 7, 59], reporting a prevalence estimate of approximately 18–22%. However, it is important to note that comparisons with prevalence rates reported in previous studies are challenged by differences in the age range of participants, the reference period of the NSSI assessment (e.g., past six or three months, past year, lifetime), and the assessment instrument (e.g., one vs. multiple items checklist). Using a similar measure and a specific age-cohort sample starting from grade 10, the results of the two-year longitudinal study by Barrocas et al. [16] showed a trend like that found in the current study, reporting a mean prevalence of NSSI of 14.8% over eight assessments, and a higher prevalence rate in Grade 10 compared to Grade 12. With respect to the Italian context, the prevalence rate reported in a previous study was higher than the prevalence rates reported here (23.6%; [26]), but the authors used a wider age range (between 14 and 19 years) and a longer reference period (last year).

Also, no significant differences were found between females and males in prevalence rates of NSSI engagement. Overall, this result is not surprising, as there is no consistency in the literature about gender-based effects [6], perhaps due to cultural differences (e.g., Western vs. Asian culture) that might influence the role of gender on NSSI [16], or methodological issues such as the different ages of participants or the assessment of one vs. multiple types of NSSI behaviors. For instance, previous studies have highlighted a robust gender difference in the behavioral methods adolescents and young adults in community samples use to engage in NSSI. More specifically, males seem to be more likely to self-hit, whereas females are more likely to cut (e.g., [61]). Given that the current study did not distinguish among NSSI methods, this could be a plausible reason explaining why no gender-based differences were observed at the descriptive level. However, it is important to outline that, based on the meta-analytic work by Bresin et al. [62], gender differences in community samples are generally very small compared to samples from clinical settings, with females scoring only slightly higher compared to males. As the authors argued, one possible explanation is that women with NSSI are more likely to seek help compared to men, thus making them overrepresented in clinical samples.

The examination of the longitudinal change of NSSI (Aim 1 of the study) allowed us to statistically test how NSSI prevalence and severity varied over time. As expected, the results revealed a negative curvilinear trend of both NSSI engagement at least once and frequency of occurrences over time, once initiated. More in detail, the probability of engaging in NSSI increased in the first part of the data collection (from T1, Grade 9, to T2 and T3, Grade 11), followed by a significant decline at T4 (Grade 12). These findings are in line with what emerged from previous secondary studies [10, 11] and provide further empirical support to recent accelerated longitudinal studies demonstrating that the prevalence rates of NSSI reach a peak during mid-adolescence and diminish afterward [12, 13]. Based on the theoretical model of NSSI development and maintenance proposed by Nock [1], this peak during mid-adolescence could coincide with an increase of intra- and inter-personal stressors that adolescents might perceive as particularly overwhelming. Also, adolescent vulnerability might be amplified by the elevated levels of impulsivity and emotional reactivity that are present during this crucial stage due to brain developmental processes [63], thus creating a need to use NSSI to better respond to and modulate their negative feelings and thoughts. Beyond this, our findings suggested that this up-and-down pattern concerned not only the probability of engaging in NSSI, and then the prevalence rates of NSSI over time, but also the frequency of NSSI instances once initiated, that first increased and then decreased. This is somewhat consistent with the study by Barrocas et al. [16] who followed a sample of 10th graders for two years across eight follow-ups (3-month intervals), finding an overall decrease in NSSI frequency of engagement over time. The fact that, in this study, both the prevalence and the severity of NSSI episodes followed a negative curvilinear trend could be a marker that both processes might be supported by the same psychological mechanisms and cognitive-emotional factors, such as the use of rumination and negative affect [64]. With this respect, Turner and colleagues [12], for instance, found that NSSI cessation several years after the first onset was associated with gradual improvements in depression, possibly reflecting a reduced tendency toward rumination and negative affect, consistently with the Emotional Cascade model [64].

Taken together, these findings indicate that, at least in community samples, adolescence is a crucial stage for NSSI, as it is during this developmental period that NSSI initiates, persists, and progressively declines. Nevertheless, although NSSI seems to have a transitional basis, the emotional and behavioral problems that young people experience as related to NSSI (e.g., depression, anxiety, lower self-acceptance) continue even several years after the behavior itself has resolved [12]. It is thus of paramount importance to understand the developmental features of this maladaptive behavior to set the basis for preventing its onset and impact on the individual long-term wellbeing.

While it is widely documented that anxiety-depression and bullying victimization are salient risk factors for NSSI [29], still limited is our knowledge about the role of temperamental EC [35, 36], above and beyond the effect of other risk factors [44]. The results of the current study (Aim 2) indicated that low EC was significantly associated with a heightened likelihood of engaging in NSSI at least once, net of the effect of anxiety-depression and bullying victimization. This finding is in line with our hypothesis and the results from previous research [35, 36], also providing further support to the hypothesis that temperamental self-regulatory abilities might play a role in determining NSSI involvement, independently of other psychosocial conditions that may co-occur [44]. However, the effect of EC only concerned the probability of engagement in NSSI, and not also the NSSI frequency once initiated. In this respect, it is noteworthy that both EC and NSSI have been recognized as important components of the clinical expression of borderline personality [31]. Thus, the significant association between EC and NSSI engagement could help to explain the high comorbidity of NSSI in adolescents with borderline personality features [35]. Also, the fact that EC typically increases from age 14 to 19 [65] could explain why NSSI decreases throughout adolescence. That is, as the capacity for EC dramatically improves due to maturation, adolescents would become more able to effortfully cope with negative emotions [66], thus reducing their need to use NSSI to manage experiences that would be otherwise perceived as overwhelming.

As for the control variables considered in the study, the results supported the significant association of anxiety-depression symptoms and bullying victimization with NSSI engagement [10, 23, 29]. Interestingly, only anxiety-depression significantly predicted NSSI frequency, meaning that, once initiated, the occurrence of NSSI episodes only depended on anxiety-depression, with higher levels predicting more frequent episodes of NSSI. Neither EC nor bullying victimization contributed to increasing the frequency of NSSI episodes. This result would corroborate theories of NSSI proposing that the urge to engage in NSSI occurs when experiencing high negative affective states, reinforced by the fact that, following the actual act of NSSI, these states may be momentarily reduced [1]. Evidence coming from ecological momentary assessments has supported this hypothesis, revealing that negative affect increases before engaging in NSSI and heightens the probability to engage in NSSI [67–70].

Finally, we found that gender was significantly associated only with prevalence rates of NSSI over time, with males being more likely to not engage in NSSI over time, compared to females. No significant associations were found between gender and the baseline probability to engage in NSSI or frequency of NSSI once initiated, as well as between gender and change of NSSI frequency over time. To our knowledge, only the study by Barrocas et al. [16], to date, has investigated the role of gender on the longitudinal trajectories of NSSI. More specifically, they examined the effect of gender on group-based trajectories of NSSI frequency, finding that boys had a higher probability to follow a moderately high and decreasing NSSI trajectory class over time, or a chronic stable trajectory class. Further research is needed to better understand if and how the probability of engaging in NSSI, as well as the frequency of NSSI occurrences, vary over time differently depending on gender.

Overall, the findings from this study provide potential implications for future research and treatment of NSSI in adolescence. In terms of future direction for research, the results suggest the importance of examining the NSSI phenomenon in both terms of presence/absence (i.e., prevalence) and frequency (i.e., severity) of the behavior, simultaneously. This would not only help to overcome the methodological issues that actually prevent scholars from making comparisons between the studies but, most importantly, it would dramatically improve our understanding and knowledge of the processes that underlie the involvement in NSSI. Drawing on this study’s results, for instance, it seems that EC abilities play a significant role in the onset of NSSI. However, once initiated, the frequency with which adolescents engage in NSSI does not depend on their levels of EC. Also, we found that the engagement in NSSI at least once and the frequency of NSSI behaviors followed a similar change trend over time. This allowed us to speculate that both might be supported by the same psychological mechanisms or cognitive-emotional factors, but further research should deepen this point. Also, future studies with larger samples could consider the specificity of the different behavioral methods for NSSI. This new perspective would extend our knowledge about NSSI characterization, also providing further insights into the association between NSSI and gender. From a clinical perspective, given the relevance of self-regulatory skills on NSSI onset, the implementation of interventions addressing skills training components, such as emotion regulation, problem-solving, or interpersonal skills is highly recommended [71], both at individual and collective levels. The DBT Skills Training for Emotional Problem Solving for Adolescents (DBT STEPS-A) [72] is an example of empirically supported psychological programs for adolescents with problems related to emotion dysregulation. DBT STEPS-A is a universal social-emotional learning curriculum derived from the Dialectical Behavioral Therapy (DBT) skills training component, including emotion regulation, reduction of impulsive behaviors, problem-solving, and building functional interpersonal relationships. The skills taught in DBT have been found to alleviate the symptoms amongst adolescent psychiatric outpatients with repetitive self-harm [73], and have been demonstrated to effectively decrease the likelihood of young individuals from non-clinical populations engaging in risky behaviors [72].

Our findings need to be considered in light of several limitations. First, it is important to note that all the study’s variables were measured using self-report questionnaires. In general, the use of a unique source of information requires caution when interpreting the results, as they could be altered due to potentially shared method variance. Second, despite the strength of the four-year longitudinal design, this study examined NSSI trajectories in a relatively short timeframe compared to the wider developmental period of adolescence. Future studies could capture the developmental course of NSSI within a longer lifespan by analyzing the change of NSSI from the beginning of early adolescence (i.e., 10 years) until the end of young adulthood (i.e., 21 years). Furthermore, it is important to note that this study, like most longitudinal studies, might have suffered from a significant attrition effect, which specifically concerned the last assessment point (i.e., the transition from T3 and T4). Although missingness was addressed in the current study by using a method that has been demonstrated to be robust for missing-data treatment in longitudinal designs (i.e., FIML), we cannot exclude that data were missing not at random, which might have biased the results. Finally, it should be acknowledged that other variables that were not considered in the current study, such as child abuse and neglect and adverse family-life experiences, might be relevant to the development of NSSI and the severity of this behavior during adolescence [29]. Future studies could investigate the potential effect of temperamental effortful control when controlling for family-based risk factors or their interplay on the onset, maintenance, and frequency of NSSI over time. Finally, it should be acknowledged that this study was not based on a probabilistic sampling procedure, which could have limited the full representativeness of the sample and, in turn, the degree to which this study’s findings can be generalized to the larger population.

In conclusion, findings from this study provide important insights into the developmental course of NSSI and point out the need for future in-depth investigations of the mechanisms that might underlie NSSI prevalence and severity throughout adolescence. Also, they underscore the notion that low temperamental self-regulatory abilities might play a crucial role in increasing adolescents’ vulnerability to engage in NSSI, over and above other risk factors. Research that considers these aspects would help design and inform interventions that can effectively prevent the development and maintenance of NSSI during adolescence, as well as the negative consequences on the individual psychological wellbeing that persist even after NSSI cessation.

### Supplementary Information

Below is the link to the electronic supplementary material.

Supplementary file1 (DOCX 17 KB)

## Data Availability

Not applicable.
