# Supplementary material for: Prevalence of engagement and frequency of non-suicidal self-injury behaviors in adolescence: an investigation of the longitudinal course and the role of temperamental effortful control
Source: Eur Child Adolesc Psychiatry. 2022 Sep 19;32(12):2399–414. doi: 10.1007/s00787-022-02083-7 (PMC10682258; doi:10.1007/s00787-022-02083-7)
Supplement: Supplementary file 1 — Supplementary file1 (DOCX 17 KB) [file 787_2022_2083_MOESM1_ESM.docx]

Table S1.

*Parameter estimates from the conditional LGC model.*

|  | | | | | Intercept | Linear Slope | Quadratic Slope | NSSI outcome |
| --- | --- | --- | --- | --- | --- | --- | --- | --- |
| Binary outcome | | | | |  |  |  |  |
| Gender (Female) | | | | | 0.00, *p* = 0.99 | -2.01, *p* < 0.001 | 2.01, *p* < 0.001 | - |
| Effortful control* | | | | | - | - | - | 0.79, *p* < 0.05 |
| Anxiety-depression* | | | | | - | - | - | -3.09, *p* < 0.001 |
| Bullying victimization* | | | | | - | - | - | -0.66, *p* < 0.05 |
|  | | | | |  |  |  |  |
| Continuous outcome |  |  |  |  |  |  |  |  |
| Gender (Female) | | | | | -0.39, *p* = 0.28 | -0.44, *p* = 0.76 | 0.42, *p* = 0.40 | - |
| Effortful control* | | | | | - | - | - | -0.18, *p* = 0.48 |
| Anxiety-depression* | | | | | - | - | - | 0.64, *p* < 0.01 |
| Bullying victimization* | | | | | - | - | - | 0.00, *p* = 0.98 |
| *Note*. Unstandardized coefficients are reported.  *Effects on NSSI were constrained to be equal over time | | | | | | | |  |
